# Genotyping-by-Sequencing for *Populus* Population Genomics: An Assessment of Genome Sampling Patterns and Filtering Approaches

**DOI:** 10.1371/journal.pone.0095292

**Published:** 2014-04-18

**Authors:** Martin P. Schilling, Paul G. Wolf, Aaron M. Duffy, Hardeep S. Rai, Carol A. Rowe, Bryce A. Richardson, Karen E. Mock

**Affiliations:** 1 Department of Biology, Utah State University, Logan, Utah, United States of America; 2 Department of Wildland Resources, Utah State University, Logan, Utah, United States of America; 3 Rocky Mountain Research Station, USDA Forest Service, Provo, Utah, United States of America; 4 Ecology Center, Utah State University, Logan, Utah, United States of America; University of Umeå, Sweden

## Abstract

Continuing advances in nucleotide sequencing technology are inspiring a suite of genomic approaches in studies of natural populations. Researchers are faced with data management and analytical scales that are increasing by orders of magnitude. With such dramatic advances comes a need to understand biases and error rates, which can be propagated and magnified in large-scale data acquisition and processing. Here we assess genomic sampling biases and the effects of various population-level data filtering strategies in a genotyping-by-sequencing (GBS) protocol. We focus on data from two species of *Populus*, because this genus has a relatively small genome and is emerging as a target for population genomic studies. We estimate the proportions and patterns of genomic sampling by examining the *Populus trichocarpa* genome (Nisqually-1), and demonstrate a pronounced bias towards coding regions when using the methylation-sensitive *Ape*KI restriction enzyme in this species. Using population-level data from a closely related species (*P. tremuloides*), we also investigate various approaches for filtering GBS data to retain high-depth, informative SNPs that can be used for population genetic analyses. We find a data filter that includes the designation of ambiguous alleles resulted in metrics of population structure and Hardy-Weinberg equilibrium that were most consistent with previous studies of the same populations based on other genetic markers. Analyses of the filtered data (27,910 SNPs) also resulted in patterns of heterozygosity and population structure similar to a previous study using microsatellites. Our application demonstrates that technically and analytically simple approaches can readily be developed for population genomics of natural populations.

## Introduction

Genetic studies of natural populations have traditionally relied on low numbers of loci to make inferences about evolutionary and demographic processes at various temporal and spatial scales. However, recent advances in sequencing chemistry, sequencing platforms, data storage, and computational processing are enabling the efficient collection of data from thousands to hundreds of thousands of loci from multiple individuals [1]. The ability to sample genomes densely at the population level is leading to a rapid radiation of analytical and bioinformatic approaches for population genomics. These approaches will greatly increase our understanding of evolutionary, demographic, and adaptive mechanisms operating in populations, and how these processes vary across the entire genome. The scales of these datasets and analyses present new challenges, including various types of chemical and technical biases, sequencing errors, and genotyping errors, all of which can be inadvertently propagated and magnified through data handling pipelines. The characterization and appropriate treatment of these biases and error sources is a critical aspect of these emerging approaches to population genomics.

One of the most exciting developments in population genomics is the development of various reduced-representation protocols, collectively referred to as Genotyping-by-Sequencing (GBS), which allow sequencing of a subset of the genome through selective amplification of restriction fragments [2]–[6]. The individual-specific oligonucleotide barcoding of these sequence libraries allows high-level multiplexing followed by bioinformatic recovery of individual identities. GBS protocols are being actively modified to optimize library and fragment sizes [7]–[10], and open-source software is becoming available for genotyping using GBS data (Universal Network Enabled Analysis Kit: UNEAK) [11]–[13]. However, the empirical effects of various library preparation and filtering techniques associated with GBS have only been explored in a very limited number of systems [7], [14]–[18]. As GBS protocols develop into mainstream tools for population-level analyses, such empirical studies of inherent errors and biases are increasingly important. In this study, we explore the effects of library preparation using methylation-sensitive restriction enzymes following Elshire et al. [10], and assess the impacts of various data filtering techniques on locus numbers, genotype assignment and population metrics.

One potentially problematic issue with GBS analyses is the large proportion of repetitive elements in complex plant and animal genomes. Sequences from repetitive regions can consume sequencing capacity without increasing information content in population analyses. The GBS approach described by Elshire et al. [10] was developed to minimize the selection of repetitive genomic regions by using a methylation-sensitive restriction enzyme (*Ape*KI). The Elshire et al. [10] technique has been used successfully in a variety of genetic mapping, population structure, and genomic selection studies in model and non-model plant species [7], [10], [12]. However, the empirical bias toward non-repetitive genomic sites has not been demonstrated in species other than a few grasses [19]. Because methylation patterns may vary among taxa, resulting in different patterns of bias, we sought to describe the patterns of genome sampling using *Ape*KI in a eudicot. We performed this analysis using tissue from the *Populus trichocarpa* clone ‘Nisqually-1’; the individual clone used to assemble the first complete annotated genome in a forest tree (genome size 485 million bp) [20]. Specifically, we sought to determine: a) whether the use of *Ape*KI following the Elshire et al. [10] protocol resulted in a bias toward coding regions relative to in silico digests (which would be agnostic to methylation), and b) whether sampling across genomic regions was relatively even. Using several biological replicates of the Nisqually-1 clone, we also sought to describe c) the frequency with which particular genome sites are sampled across replicates, and d) the relationship between sequencing depth and genome sampling intensity.

Another issue with GBS analyses is accounting for variation in coverage depth and sequence read quality in individual genotyping and population allele frequency estimation [16], [21]–[24]. Two general approaches are emerging for the handling of GBS data. One approach is to use all the data regardless of quality and then to use Bayesian probabilities based on sequence quality and depth throughout the analytical pipeline [24]–[26]. In this method, a fixed genotype is not strictly assigned to an individual, but their probabilities are considered throughout any downstream analyses. An alternative is to filter out data of low quality and loci of low read depth before performing population genetic analyses. This approach has the advantage of being less computationally intensive since genotypes are assigned, and the data substantially reduced early in the analytical pipeline. Here we assess how filtering loci using this second approach affects both genotype assignment and population structure metrics. We explored three different filtering strategies based on specific thresholds for minor allele frequencies and read depth. We compare the behavior of these filters with respect to the number of loci retained, estimates of individual and population-level heterozygosity, Hardy-Weinberg equilibrium, and measures of population subdivision.

To assess the impact of various data filtering strategies, we chose trembling aspen (*Populus tremuloides*) because of its close relationship to *P. trichocarpa* [27] and its remarkably broad longitudinal and latitudinal distribution [28]. The distribution of this species across many different ecological gradients makes it an excellent candidate for future population genomic studies of adaptation and climate change effects. *Populus tremuloides* consists of two major genetic clusters: one in Canada and the northern U.S. (N cluster) and another in the western U.S. (SW cluster) [29]. This allows us to assess the effectiveness of GBS to detect population differentiation at multiple scales. The samples used in this study come from 6 populations representing both major clusters, and the output from various filtering strategies were compared with the output from previous microsatellite analysis of the same populations [29].

## Materials and Methods

### Sample acquisition and library preparation

#### Plant material and genomic DNA isolation

For assessment of gene sampling in GBS, we obtained leaf material from a clone of Nisqually-1, the *P. trichocarpa* genotype used for genome sequencing as part of the *Populus* genome project [20]. For population studies, we sampled *P. tremuloides* genets from each of 6 populations, including four from the N cluster: FLFL (Flin Flon, Manitoba, Canada, n = 16), HSPQ (Havre-St.-Pierre, Quebec, Canada, n = 20), MI (Ontonagon County, Michigan, USA, n = 20), SFQ (Saint-Felicien, Quebec, Canada, n = 20), and two populations from the SW cluster: KFO (Klamath Falls, Oregon, USA, n = 12), and WWA (Kittitas County, Washington, USA, n = 19) [29]. Dried leaf tissue was ground in a Tissuelyser II (Qiagen Inc., Valencia, CA) with tungsten carbide beads. DNA extraction was performed using a Qiagen DNeasy Plant kit (Qiagen Inc., Valencia, CA). The final DNA product was eluted twice from each column into 60 µL and 30 µL of AE buffer, respectively. The genomic DNA was quantified using a Qubit fluorometer (Invitrogen, Carlsbad, CA).

#### Library preparation and high-throughput sequencing

For both *P. trichocarpa* (Nisqually-1) and *P. tremuloides* samples, genomic libraries were prepared following Elshire et al. [10] with the methylation-sensitive restriction enzyme *Ape*KI and custom adapters and barcodes. For *P. trichocarpa*, 16 uniquely barcoded GBS genomic libraries (replicates) were constructed using the same DNA extraction. For *P. tremuloides*, uniquely barcoded *Ape*KI libraries consisted of 152 samples representing 107 individuals. Forty-five of the samples were replicates: 18 individuals run in duplicate, and three individuals run in triplicate, all from common DNA extractions. Illumina high-throughput sequencing was performed at the Vanderbilt University Medical Center, on an Illumina HiSeq 2000 using 100 bp single-end indexing runs. The samples were sequenced across two Illumina lanes. Base calling was performed in Casava v1.8 (Illumina, San Diego, CA).

#### Read sorting

For both the Nisqually-1 and *P. tremuloides* samples, index (barcode) deconvolution was done using a custom Perl script to sort each of the GBS barcoded samples into separate fastq files. The individual raw read files (fastq) were imported into CLC Genomics Workbench (v4.9; CLC Bio, Cambridge, MA) and further processed for quality and length (trimmed using quality scores with a limit set to 0.05; discarded reads less than 30 bp). The enzyme recognition sequence was removed from the 5′ end of each raw sequence read after quality trimming.

### Assessment of coding regions sampled in reference genome

Each of the 16 Nisqually-1 replicated libraries, consisting of single-end trimmed reads, was individually aligned to the *P. trichocarpa* v2 assembly (∼403 Mb arranged in 19 chromosomes, assembled into 2518 scaffolds; Phytozome v8.0) using CLC Genomics Workbench (v4.9; CLC Bio, Cambridge, MA). The maximum gap and mismatch count were set to 2, and insertion and deletion costs were set to 3, with a minimum contig length of 200 bp. Length fraction and similarity parameters were set to 0.6 and 0.8, respectively. We used the above assembly to determine whether each GBS read was or was not within an annotated gene on the *P. trichocarpa* genome.

To determine whether observed GBS loci occurred randomly across the *Ape*KI sites known to occur in each of the genomic scaffolds, we conducted a chi-square test comparing the number of observed vs. expected GBS loci. We calculated the expected number of GBS loci in each scaffold by multiplying the total *in silico* number of *Ape*KI-containing genes (40,666) by the overall proportion of these genes that was sampled by GBS (34,750 or 85%). Only scaffolds containing more than five *Ape*K1 sites (83 of a total of 998 *Ape*KI-containing scaffolds) were used in this analysis.

To explore the effect of increasing read depth on genome coverage, we sequentially added individual *P. trichocarpa* (Nisqually-1) replicates, and counted the accumulating total number of sites sampled. We randomly ordered the replicates and averaged the effect over 1000 permutations. We acknowledge that accumulating replicates in this fashion is not technically equivalent to reducing the level of multiplexing in a single run, given that different sequencing runs have different error profiles. Our goal here was to estimate the effect of multiplexing level using a proxy.

### Assessment of data filtering strategies in *P. tremuloides*

#### UNEAK pipeline

For *P. tremuloides* samples, single nucleotide polymorphism (SNP) genotypes were assigned using the UNEAK (Universal Network Enabled Analysis Kit) filter [12] with default settings. This pipeline was designed for taxa for which no reference genome is available, which is currently the case for *P. tremuloides*. Using Illumina sequence reads, the network filter in UNEAK trimmed reads to 64 bp to minimize the effects of sequencing error, and enabled efficient storage of data in bit format. Identical reads are then collated as haplotypes (referred to as tags by Lu) [12]. Haplotype pairs differing by a single nucleotide were retained as SNPs. Any SNP with a read depth >127 was removed, to eliminate loci that have multiple genomic copies. Those SNPs with a minor allele frequency <0.05 were removed to minimize the impact of sequencing errors [12].

#### Post-UNEAK pipeline

Prior to the application of alternative filtering strategies, we applied two functions that removed all SNPs (rows) and then samples (columns) containing 90% or more ‘N’ values (indicating that neither allele is designated). These Ns represent individuals where the allele cannot be called from the sequence reads. This is either because no read is available at this site (for this individual) or the sequence quality is too low to call. Because we are attempting to retrieve SNPS for population genetic analysis, we apply this early filter to remove loci and individuals that contain very low levels of information prior to further filtering.

**Figure 1:**
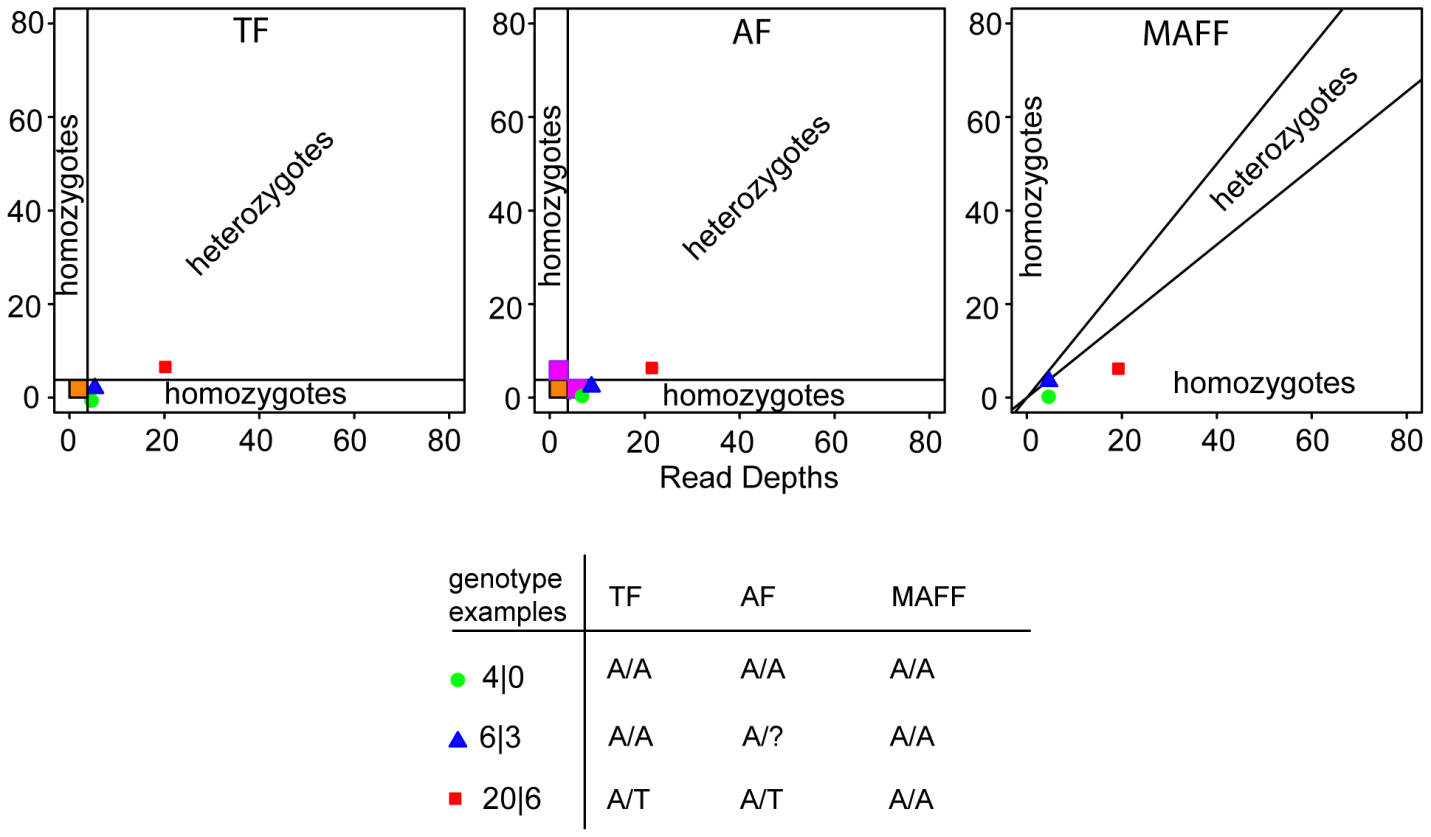
Graphical representation of our three filtering schemes, with examples of how some genotypes are interpreted. Orange boxes denote alleles that are filtered (converted to ‘N’) and pink denotes alleles that are converted to ambiguous (‘?’).

We used custom Python scripts (available at https://github.com/schimar/gbs) to read the UNEAK output files and to apply three different filters (Fig. 1). The Threshold Filter (TF) discards all SNPs having a total read depth of less than four (default threshold value). Using the TF, SNPs with 4–7 (and >7) identical reads are scored as a homozygote. A potential problem with this approach is that a heterozygote call would require four reads of each allele, but a homozygote call requires only four reads. I.e. to be ‘fair’ we should require 2×4 = 8 reads to call a homozygote. Our Ambiguity Filter (AF) handles this by assigning an unknown (‘?’) to the second allele in cases where the read depth is 4–7. Thus, a total of 8 reads containing an ‘A’ at a SNP locus would be assigned a homozygous genotype ‘AA’, whereas a total of 4–7 reads containing ‘A’s would be assigned a genotype ‘A?’. The Minor Allele Frequency Filter (MAFF) assigns homo- or heterozygous genotypes based on the allele frequency of the minor allele, with a threshold of 0.45. Therefore, the MAFF does not discard alleles from genotypes based solely on low read depths. After filtering, we repeated the exclusion of SNPs with 90% or more ‘N’ values. At this point, we have a matrix of individuals x SNP genotypes. From this we counted the total number of cells that were homozygous, heterozygous, and ambiguous.

#### Genotype Mismatch rates

For each filtering strategy, we assessed the frequency of allelic mismatches across replicated samples. Thus, if a genotype is ‘GA’ in one replicate and ‘GG’ in the other this counts as one difference (a single mismatch), whereas ‘GG’ versus ‘AA’ is two differences (a double mismatch). Ambiguous alleles (coded as ‘?’) and unknown (‘N’) alleles were not included as mismatches. We report mismatch rates as the proportion of alleles differing among all alleles in the matrix (i.e., 2 x the number of SNPs). We anticipated that low read depths would contribute to mismatches by increasing allelic dropout and by increasing error rates in base calling. To test this hypothesis we compared read depths between replicated pairs in which one sample was a heterozygote and the other was a homozygote (i.e. allelic dropout) and assessed the read depth difference between each pair using a Wilcoxon signed rank test. Further, we tested the read depth difference between mismatched homozygous samples and matched homozygous samples, using a Mann-Whitney test.

#### Filtering effects on population parameters

For population analyses, we represented each replicated individual once, choosing the individual with the greatest overall read depth. For the unfiltered data, as well as those from all 3 filters, we used the Python HWE exact test [30] to calculate HWE probabilities for each SNP and population. For each filter, locus-specific HWE probabilities were pooled across populations, and results summarized as violin plots. One of the six populations (KFO) was excluded from this analysis because of low sample numbers for estimating p(HWE).

Using the output from each filter, we calculated F_ST_ values among populations, along with mean observed heterozygosity (Ho) and estimated heterozygosity (Hs) for each population, averaged across populations, using the R packages adegenet [31], [32] and hierfstat [33]. Ambiguous alleles were treated as missing data in these analyses. In previous population genetic studies of aspen, pronounced subdivision was detected between northern (including FLFL, HSPQ, MI and SFQ) and western populations (including KFO and WWA) using microsatellite markers [29]. To test whether this same pattern was evident in the GBS data, we explored relative patterns of F_ST_ in SNPs and msat loci from the same populations.

## Results

SNP data for this project are publicly available, in hapmap format, from the Dryad repository: http://dx.doi.org/10.5061/dryad.2cs4g.

### Assessment of coding regions sampled by GBS using *Ape*KI

We used the *P. trichocarpa* genome to study the genomic patterns and distribution of regions captured by the GBS technique. There are 462,987 *Ape*KI in silico restriction sites in the *P. trichocarpa* (Nisqually-1) genome. Of those, 212,376 (45.9%) are within annotated gene boundaries. By contrast, in our 16 GBS replicates of *P. trichocarpa*, sequence reads were recovered from a mean of 125,022 ApeKI sites (loci) per replicate (27% of in silico restriction sites) (s.e. 6,363), of which 87,202 (69.8%) (s.e. 4,289) were within annotated genes. Thus, our use of *Ape*KI consistently captured loci within annotated genes more frequently than one would expect by chance alone (*P*<<0.005 according to a simple contingency test).

Pooling results from all 16 replicates, we found that 84% of all annotated genes in the *P. trichocarpa* genome were represented in the GBS library. The number of annotated genes within genomic regions (scaffolds) sampled by GBS was not significantly different from a random subset of the genes present (*P* = 0.61), indicating that GBS sampled these regions evenly.

**Figure 2:**
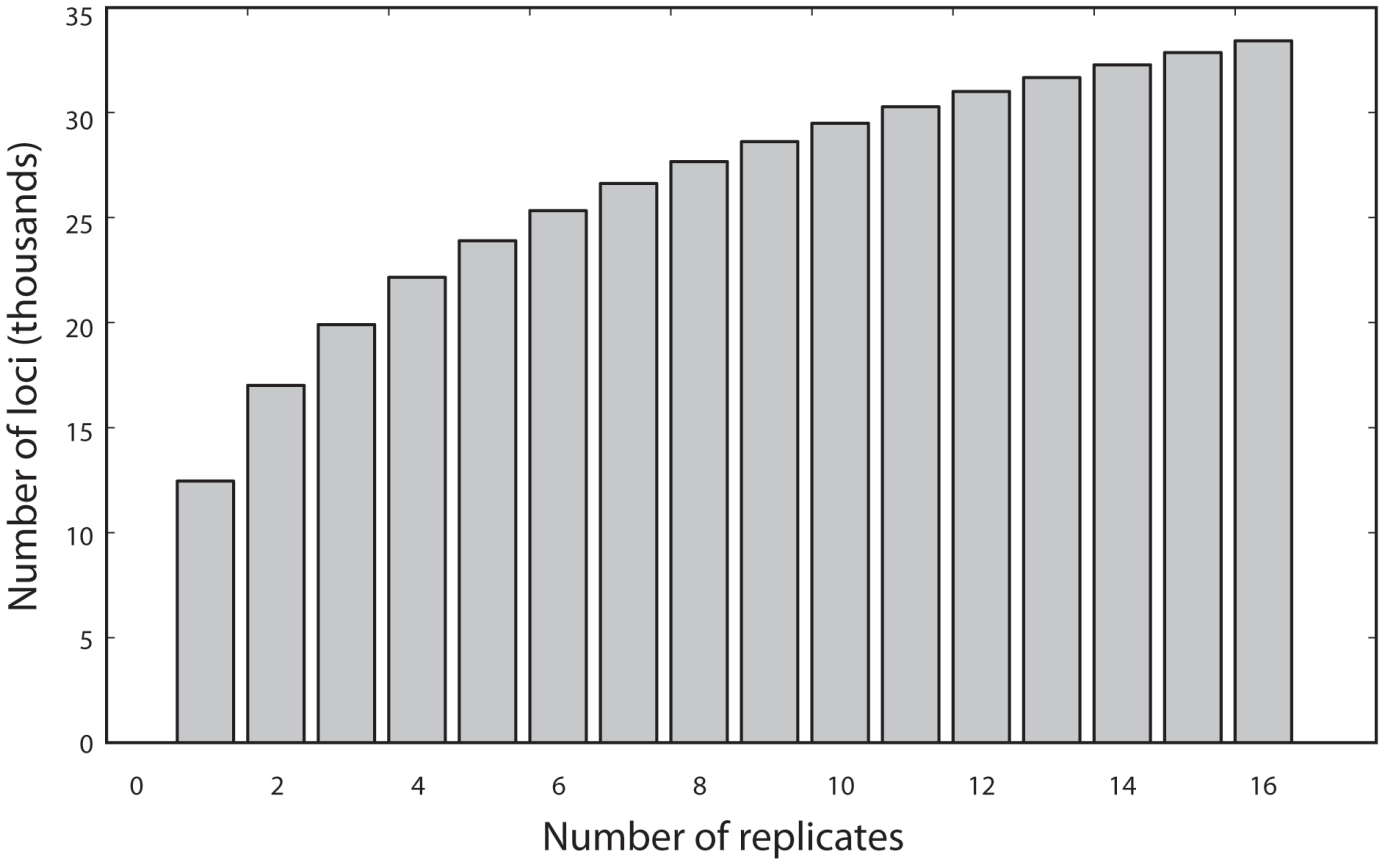
Effect of the number replicates on the number of unique regions (loci) captured by GBS in *P. trichocarpa*. We performed 1000 permutations, each with a random order of adding the 16 replicates.

Across the 16 *P. trichocarpa* replicates, we detected 334,158 loci. On average, 26% of these loci were detected in only one of the 16 replicates, and 9.6% were detected in all 16 replicates. The cumulative distribution of locus numbers over replicates (Fig. 2) indicated that although at 16 replicates we are approaching an asymptote, the graph is not yet leveled. Thus, although we can detect more loci by reducing the level of multiplexing, the returns are diminishing.

### Assessment of SNP filters in *P. tremuloides* populations

From the 152 barcoded samples in our study, we obtained a total of 23 billion bp of sequence from 289 million reads, for an average of 1.9 million sequence reads per sample. After removal of SNPs with a minor allele frequency <0.05, we had 160,183 SNPs for further analysis. The mean number of reads per individual was 45,149 (s.e. 850), and the mean number of reads retained by UNEAK per sample was 220,872 (s.e. 6674). After removing samples and SNPs with more than 90% ‘N’, the dataset consisted of 108,530 SNPs representing 101 individuals (FLFL: 16; HSPQ: 20; KFO: 6; MI: 20; SFQ: 20; WWA: 19) and 39 replicates. This dataset was subjected to the three different filters (TF, AF, MAFF). The MAFF dataset retained all 140 samples and 74,159 SNPs, whereas TF and AF datasets each retained 140 samples and 27,910 SNPs. The total numbers of ‘NN’ (unassigned) genotypes for each filter was 35628 (no filter and MAFF) and 11,929 (AF and TF).

**Figure 3:**
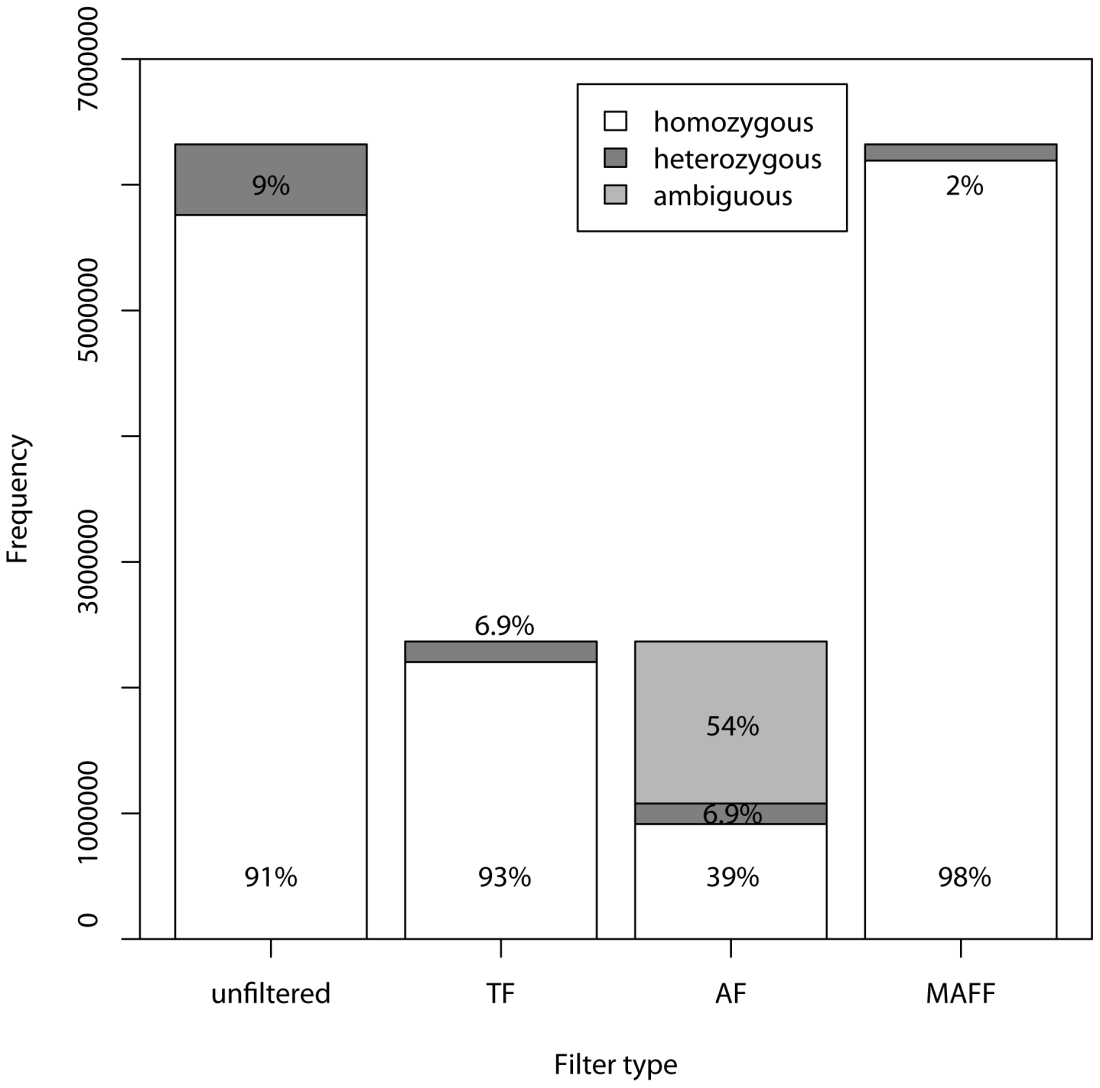
Pooled distribution of genotypes for unfiltered and filtered SNP data for *P. tremuloides*.

The proportions of homozygous and heterozygous genotypes at each SNP are shown for each filter in Fig. 3. Prior to application of our filters, 26% of the SNP/individual combinations were assigned a genotype, of which of 9% were heterozygous and 91% were homozygous SNPs. The proportion of heterozygous genotypes was reduced by all filters (to 6.9% for both TF and AF and 2% for MAFF). This result indicates that all three filters are removing alleles contributing to observed heterozygosity.

**Figure 4:**
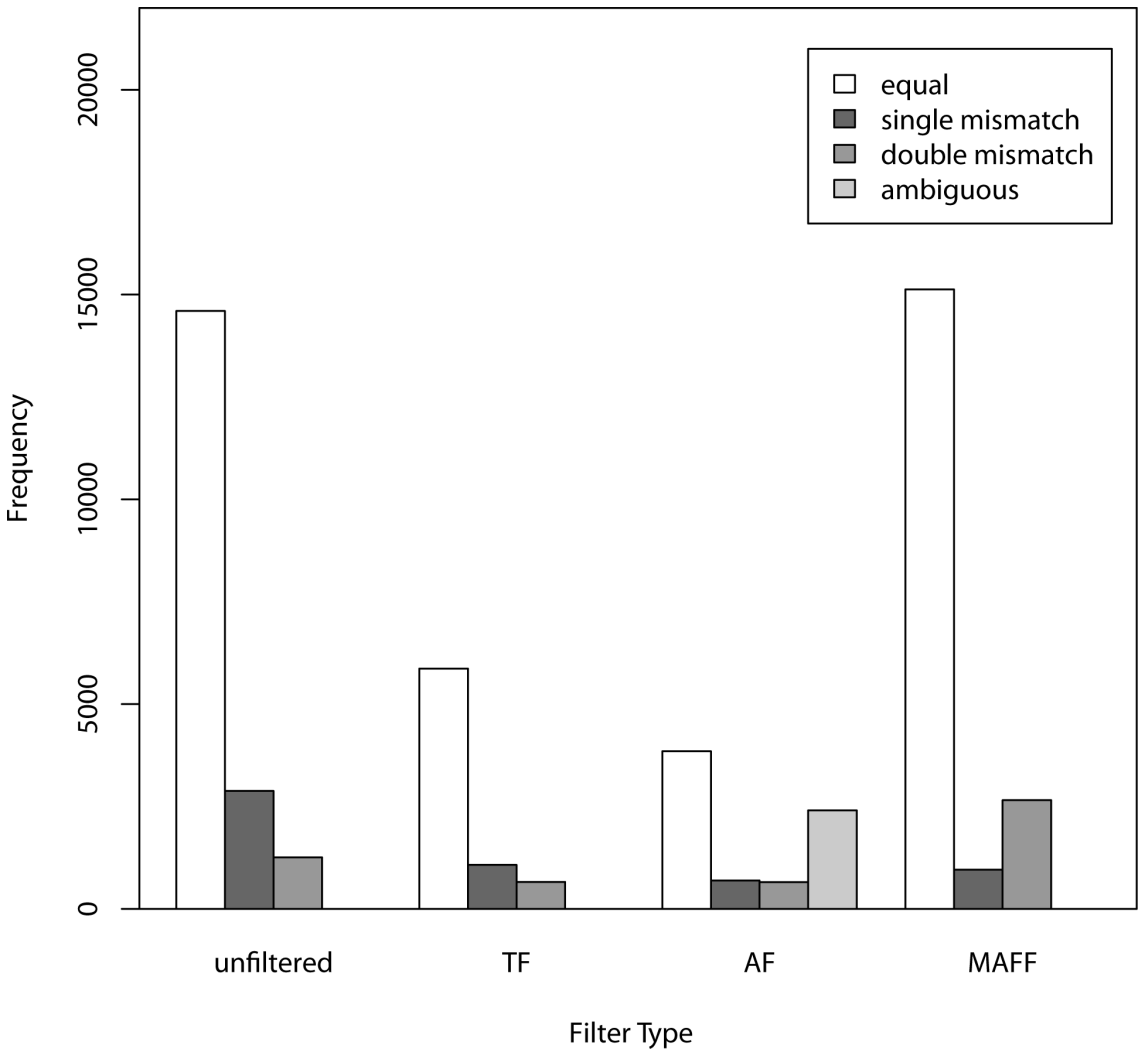
Numbers of matches, single mismatches (e.g., AA versus AT), double mismatches (e.g., AA versus TT) and ambiguous differences between replicates for unfiltered and filtered SNP data. Comparisons that include (‘NN’) genotypes are not included here.

Mismatches across replicated samples at each SNP were calculated for each filtered data set (Fig.4). In the unfiltered dataset, the mismatch rate was 1.8%, TF was 1.95%, AF was 1.65%, and MAFF was 1.97%. Mismatch rates did not differ greatly across populations (data not shown), but there was variance across replicate pairs compared, with mismatches ranging from 0.04% in the AF to 6.3% in the unfiltered data. Overall, it appeared that filtering, especially the AF, reduced the mismatch rates. Mismatches in each category are shown in Fig 3. With unfiltered data, in mismatched pairs involving allelic dropout in one sample, the homozygote had the lowest read depth in 79.1% of cases. The average read depth in these mismatches was 4.15 for the homozygote and 7.40 for the heterozygote (p<<0.001). In mismatched pairs involving two homozygotes, the average read depth (pooling across all replicates) was 1.21, while the average read depth for matched homozygote replicates was 2.70 (p<<0.001).

**Figure 5:**
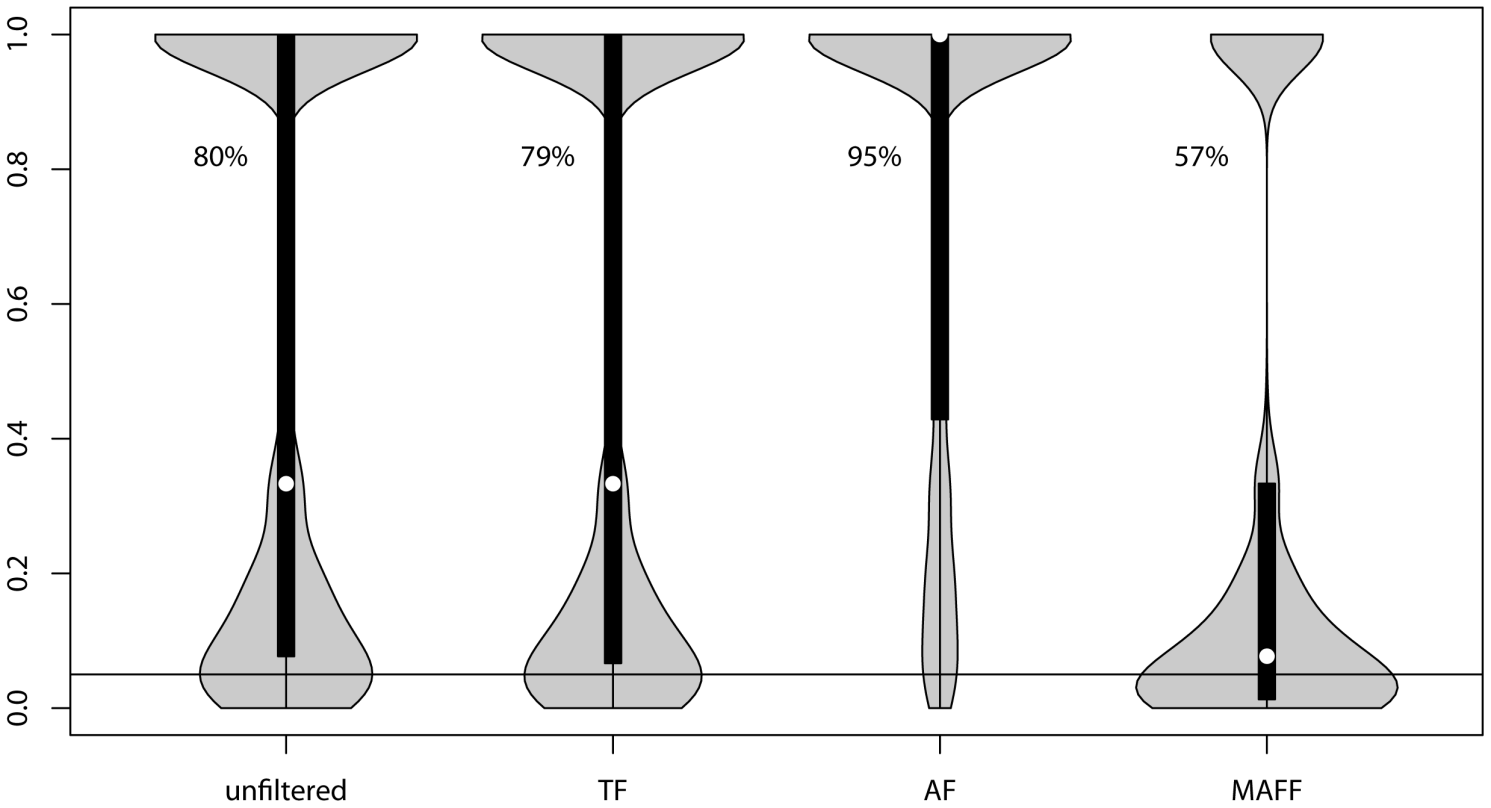
Violin plot for pooled results of Hardy-Weinberg exact testing for each SNP per population. Tests for all populations have been pooled for each filter (x-axis) and probability-values of the exact test (y-axis). The proportions of tests with probabilities above 0.05 (horizontal line) are shown for all 4 filters. White dots show the median, bottom and top of the boxes show lower and upper quartiles, respectively, and “fins” illustrate the density of observations across the entire range of probability values. Note that the numbers of tests differ for each filter (see text).

The distribution of population- and SNP-specific HWE probabilities, averaged across populations, is presented for each filtering approach (Fig. 5). The differences among the filters were similar across all populations (data not shown). In all cases, including no filtering, there was a bimodal distribution of HWE probability values. The MAFF had the lowest percentage of HWE probabilities >0.05 (57%), whereas AF had the highest (95%). This increase using the AF was due to a reduction in SNPs with observed heterozygote deficiencies relative to HWE expectations (Table 1).

**Table 1:** Mean observed heterozygosity (H_O_), mean expected heterozygosity (H_S_), F_ST_ across all populations, and mean F_ST_ between pairs of populations of *P. tremuloides* between and within the two major geographic regions in North America.

Data source	H_o_	H_s_	F_ST_	Mean F_ST_ between regions	Mean F_ST_ within regions
Unfiltered	0.0736	0.2627	0.0528	0.1585	0.0175
TF	0.0575	0.2277	0.0497	0.0002	−0.0011
AF	0.6361	0.5033	0.0120	0.0395	0.0043
MAFF	0.0188	0.2608	0.0523	0.1591	0.0169
Msats	0.7150	0.7270	0.0730	0.0978	0.0096

The patterns of population differentiation were not dramatically impacted by the choice of filter sets. In all cases, the average pairwise F_ST_ value between pairs of populations was greater between previously described genetic groups (N and SW clusters) than within each of these groups. These patterns were consistent with results from the same populations using 8 nuclear microsatellite loci. A ten-fold difference in pairwise F_ST_ values between vs. within major groups was noted with microsatellite data, the unfiltered, the AF, and MAFF data (Table 1).

## Discussion

The genus *Populus* is rapidly becoming a model study system for the examination of several aspects of forest tree biology [34] and adaptation to climate change [35]–[38]. One of the benefits of working with *Populus* is that the genus includes *P. trichocarpa*, which was the first tree species to have its genome sequenced and assembled [20]. This annotated genome provides a wealth of resources for further study in the genus [39]. *Populus tremuloides* (aspen), for example, has the broadest geographic range of any North American tree species [40] and has tremendous ecological, economic, and aesthetic value, particularly in the western portion of its range [41]. Thus, *P. tremuloides* lends itself to studies of local adaptation and evolutionary history, tractable by GBS.

Here we explored two aspects of using GBS in *Populus* species: the bias toward coding regions when using GBS with a methylation-sensitive restriction enzyme (in *P. trichocarpa*), and the effect of various data filtering strategies on population data (in *P. tremuloides*).

### Assessment of coding regions sampled by GBS using *Ape*KI

When we examined our data relative to the fully sequenced *P. trichocarpa* genome (Nisqually-1), we found that *Ape*KI increases sampling of annotated genes relative to that expected by chance alone. Previous studies have shown that *Ape*KI preferentially cuts at nonmethylated sites [42], which in most eukaryotic genomes are more likely to be coding regions [43]. This bias occurs even though not all methylation occurs in noncoding regions and the methylation preferences of the enzyme are not strict. The *Populus* genome is relatively small (410 Mb) [20] so a relatively large proportion of the genome is coding. Therefore, we would expect that in a larger genome the selection for coding regions might be even more pronounced. Thus, for researchers interested in population genomics specifically of coding regions (such as selection or association studies), *Ape*KI seems to be an ideal choice. Concomitantly, if questions require information about all genomic regions, irrespective of coding status, then a different endonuclease should be used.

The distribution of GBS loci (regardless of the presence of SNPs) of the *P. trichocarpa* genome assembly suggests that the regions captured by the GBS technique are randomly distributed across the scaffolds, although the large size of many of the scaffolds may obscure some within-scaffold clustering. Thus, we have good reason to believe that SNP loci captured by GBS (using *Ape*KI) provide a representative sample of the genome.

Of the approximately 410 Mb in the *P. trichocarpa* genome assembly [20], about 11% of the genome would be sampled if all *Ape*KI sites were captured by GBS. This value is 8% when pooling all 16 of our *P. trichocarpa* replicates, and 3% for any one replicate. These percentages would drop correspondingly for larger genomes, and they can be modified by controlling read depth via the level of multiplexing.

### Assessment of data filtering strategies in *P. tremuloides* populations

#### Filtering of *P. tremuloides* SNP data

In any research project that entails a sampling of variables, it is ideal to retain those variables that are informative for the questions at hand. In the case of population genetics based on allele frequency estimates, informative loci are those for which genotypes can be assigned most accurately. Thus, our goal in filtering was to retain as many of those SNP loci as possible. In our GBS study of *P. tremuloides*, the method of filtering had a large effect on some downstream analyses, particularly those influenced by homozygosity. Determining which filters are retaining useful data and which filters are generating artifacts can be difficult. Fortunately, we have independent data for *P. tremuloides* indicating that populations are generally in HWE [29], [44]–[46], as would be expected for a wind-pollinated tree species. Unfiltered SNPs resulted in the inclusion of many that were not in HWE, most of which were deficient in heterozygotes. A simple threshold filter (TF) did not improve this situation appreciably, nor did a filter that evaluated SNPs based on the allele ratios (MAFF). The AF appeared to retain more SNPs in HWE, suggesting that this filter may result in the most accurate estimate of heterozygosity. The AF filter assigns ambiguous alleles depending on the read depth of the second allele. Therefore, we speculate that this filter results in more accurate genotyping of the SNPs retained. Determining whether this filter is generally optimal will require more data from more species. Also critical is that, in general, a more refined filter may result in a higher proportion of useful SNPs, but it will usually result in overall fewer loci, a trade-off that needs to be addressed specifically in each study. Combining multiple filtering steps should also be explored as a means of obtaining more accurate estimates of genotype.

#### Effect of filtering strategies on population parameters

Populations of *P. tremuloides* tended to show only low levels of differentiation (Table 1). However, using GBS, we detected two genetically distinct clusters of aspen populations; the same clusters that have been detected using microsatellite data [29]. Pairwise F_ST_ values using different marker types and different loci are difficult to compare directly, since they can be influenced by locus-specific polymorphism [47]. However, we suspect that with the MAFF and TF filters, as well as with unfiltered data, many more SNPs were retained in which read depths were too low to detect another allele, thereby overestimating homozygosity. The values of both observed and expected heterozygosity using microsatellites exceeded values from all other filter types, a result expected due to high allelic richness.

#### Assessment of genotyping error

Errors and biases are not new to genotyping. Prior to genotyping based on reduced representation libraries, scoring errors [49] and errors associated with Sanger sequencing and PCR were recognized [48], [50], although routine assessment of error rates has not become common [15], [51]–[53]. Given that next generation sequencing (NGS) does not entail manual scoring of genotypes, which can suffer from subjectivity [49], using automated clustering and filtering schemes allows more objectivity, both in how we choose the markers for genotyping and in actually genotyping them. However, automated genotyping, clustering, and filtering techniques can also result in systematic biases and a high frequency of errors, particularly errors related to coverage depth, as demonstrated here.

Because we did not know the true genotype for each SNP for each individual, we assessed genotyping error indirectly by comparing replicated samples, and reporting mismatch rates. Overall, the AF had the lowest mismatch rates, consistent with its behavior in population genetic analyses. The source of mismatches is unknown, but our analyses suggest that higher mismatch rates among replicates are associated with low read depth. We expect that low read depth would cause allelic dropout in heterozygotes and increased base-calling error in both homozygotes and heterozygotes. Determining the factors that contribute to mismatches can help researcher to minimize error rates, although methods to assess errors in NGS studies are not consistent [48], which can make it difficult to compare among studies. Reducing error rates is particularly critical for some types of studies, e.g. those that involve assessment of parentage, individual identity, or locus-specific effects. Thus, understanding the effect of different data filters (of any type) on error rates is an important component of working with NGS data.

#### Tradeoffs between depth and coverage

For a taxon with no reference genome, any exploration into population genomics requires a complex assessment of how to multiplex samples optimally. The strategy chosen will ultimately affect how many SNPs can be used. But the amount of data can also be controlled by the use (or lack) of filtering systems. In general, we expect that filters that are more stringent will retain fewer usable SNPs, as we noted in our results (Fig. 3). Buerkle and Gompert [24] advocate using the maximum number of SNPs (no strict filtering), retaining those even with minimal coverage: as low as 1 read per SNP per individual. This approach is particularly appropriate for studies that examine the variance in population parameters (such as F_ST_ outlier analyses) across SNPs, where maximizing loci is critical. However, for basic questions of population structure, filtering to retain informative data might be a more efficient strategy. Thus, depending on the organism and study question, it may well be worth assessing the optimal level of multiplexing for GBS, to control read depth and number of SNPs. Note that even for the small *Populus* genome, it appears that we sampled a small proportion of available SNPs with our level of multiplexing. Additional SNPs could be sampled if we reduced the level of multiplexing. However, the approach to an asymptote (Fig. 2) suggests diminishing returns. More importantly, the level of multiplexing we used for *P. tremuloides* was still sufficient to detect over 100,000 polymorphic SNPs. Even in the *P. trichocarpa* replicates, where only 9.6% of the loci were found among all replicates, these amounted to 32,080 loci, which would certainly be sufficient for population-level analyses of gene flow patterns, even with moderately low levels of polymorphism. To increase the overall read depth and level of overlap across samples further, it would be necessary to reduce the level of genome sampling. This can be achieved through various approaches including size selection of genome fragments [14].

## Conclusions

Several versions of NGS applications are becoming available for the study of population genomics, along with a variety of approaches to analyzing the resulting large-scale data. As these new techniques emerge, assessment of the impacts of various methods for sample processing, reduction and selection of genome representation, and compiling sequence data into population-scale genotypic data are becoming an important developmental phase [54], [55]. Here we apply a technically simple NGS approach (GBS) to samples from natural populations, present an assessment of how the genome of a related species is sampled with this technique, and explore an analytical framework that provides simple genotype data that are ready for traditional population genetic analyses. We demonstrate that the use of the restriction enzyme *Ape*KI results in a bias for coding regions, even in a species that is not closely related to the species in which the method was first developed (e.g. *Zea mays*) [10]. This finding confirms the utility of GBS for analyzing differences in coding regions at the population level (e.g. for association studies). Our analysis of replicates provides insights about the trade-off between multiplexing depth and genome coverage. We also present several different data filtering approaches and show that the choice of approach can have a pronounced effect on error rates and on population parameters such as allele frequencies and HWE. We encourage researchers who are shifting to NGS population approaches to explore genotyping error rates and allele frequency distributions to understand better the potential biases that accompany various data filtering methods.
